# Electron tomography unravels new insights into fiber cell wall nanostructure; exploring 3D macromolecular biopolymeric nano-architecture of spruce fiber secondary walls

**DOI:** 10.1038/s41598-023-29113-x

**Published:** 2023-02-09

**Authors:** Dinesh Fernando, Michael Kowalczyk, Pablo Guindos, Manfred Auer, Geoffrey Daniel

**Affiliations:** 1 Department of Forest Biomaterials and Technology/Wood Science, Swedish University of Agricultural Sciences (SLU), 756 51 Uppsala, Sweden; 2 Division of Molecular Biophysics and Integrated Bioimaging, Department of Cellular and Tissue Imaging, Lawrence Berkeley National Laboratory (LBNL), 1 Cyclotron Road, Mail Stop Donner, Berkeley, CA 94720 USA; 3 School of Engineering, Pontificia Universidad Católica de Chile, Vicuña Mackenna 4860, Santiago, Chile; 4 National Excellence Center for the Timber Industry (CENAMAD), Pontificia Universidad Católica de Chile, Vicuña Mackenna 4860, Santiago, Chile; 5 Department of Biomedical Engineering, School of Biological Sciences and Medical Engineering, Southeast University, Nanjing, China

**Keywords:** Cell wall, 3-D reconstruction

## Abstract

Lignocellulose biomass has a tremendous potential as renewable biomaterials for fostering the “bio-based society” and circular bioeconomy paradigm. It requires efficient use and breakdown of fiber cell walls containing mainly cellulose, hemicellulose and lignin biopolymers. Despite their great importance, there is an extensive debate on the true structure of fiber walls and knowledge on the macromolecular nano-organization is limited and remains elusive in 3D. We employed dual-axis electron tomography that allows visualization of previously unseen 3D macromolecular organization/biopolymeric nano-architecture of the secondary S2 layer of Norway spruce fiber wall. Unprecedented 3D nano-structural details with novel insights into cellulose microfibrils (~ 2 nm diameter), macrofibrils, nano-pore network and cell wall chemistry (volume %) across the S2 were explored and quantified including simulation of structure related permeability. Matrix polymer association with cellulose varied between microfibrils and macrofibrils with lignin directly associated with MFs. Simulated bio-nano-mechanical properties revealed stress distribution within the S2 and showed similar properties between the idealized 3D model and the native S2 (actual tomogram). Present work has great potential for significant advancements in lignocellulose research on nano-scale understanding of cell wall assembly/disassembly processes leading to more efficient industrial processes of functionalization, valorization and target modification technologies.

## Introduction

Plant fibers (i.e. lignocellulosic biomass) have served for humankind for centuries in various ways and continue to play a major role in the future world while offering the largest repository of renewable carbon biopolymers (i.e. cellulose, lignin) in our biosphere^1,2^. Lignocellulosic biomass (e.g. xylem fibers from forest trees) has received an unprecedented global demand in recent decades due to the “sustainable-/bio-based society” concept and circular bioeconomy paradigm echoed, in particular across Europe^3–6^. Xylem (wood) fibers have a tremendous potential for fostering the concept contributing to a plethora of industrial sectors ranging from traditional pulp and paper, construction materials, textiles etc. to modern biorefineries, bioenergy/biofuel, biocomposites, medical-, electronics and automobile industries^3–5,7^. Wood fibers have cell walls that are highly complex and composed primarily of cellulose, hemicellulose and lignin biopolymers arranged in a hierarchical structure. However, although the gross chemistry and basic morphology of fiber cell walls is well documented (i.e. using conventional 2D imaging and traditional chemical analysis), there is still an extensive debate on the “true” structure of fiber cell walls^8–11^.

The wood fiber cell wall is characterized by lignified primary (thin) and secondary (thick) walls with the latter composed structurally of three characteristic layers (S1, S2 and S3) constituting the major part of the xylem and lignocellulosic biomass. However, the structural organization of cell wall polymers is rather complex and there is still a gap in our understanding on both its structural complexity and the structure–function relationship at nano-meter level^10^. Despite structural and chemical variations between different cell wall layers, the dominant secondary S2 layer of wood fiber walls is normally composed of three major biopolymers (cellulose, hemicellulose and lignin) molded into a complex, heterogeneous “3D biopolymer nanocomposite”. Cellulose (45–50% of dry weight) is united into microfibrils (MF) of ca 2–4 nm diameter with crystalline and amorphous regions and can exist as single or aggregate assemblies of MFs (also known as macrofibrils with reported diameters of 10–60 nm)^8,12–15^. Hemicelluloses (20–35% of dry weight) are short and branched chains composed of both homo- and heteropolymers^14,16,17^. Lignin (15–32% of dry weight) is the second most abundant plant biopolymer after cellulose, and is considered an aromatic heteropolymer forming amorphous 3D network^16,18^. The scaffold of cellulose (β-1-4-glucose) MFs in fiber secondary walls are thought surrounded by hemicellulose (mannose, xylose), whereas lignin is thought to mostly fill the gaps between cellulose MFs and/or MF aggregates^8,19^. Both hemicelluloses and lignin are widely known as ‘matrix polymers’ (or matrix biopolymer) of fiber wall.

The ‘filament-like’ MFs in mature xylem S2 are reported to wind around the cell in helical fashion at an angle of 5–20° relative to the long fiber axis, which is known as the microfibril angle (MFA). However, depending on cell type, position within the tree trunk, within the same and between species and even among individual fibers, the MFA can vary considerably, which in turn has a profound effect on the properties of wood, fibers and their products such as stiffness^20,21^. Softwoods (coniferous trees) like Norway spruce normally have a high cellulose and lignin content with lower amounts of hemicellulose composed primarily of galactoglucomannan (GGM) and lesser glucuronoxylan^16^. Nonetheless, precise description and dimensions of macromolecular structures and biopolymer 3D arrangements/distribution particularly along the MF axis (i.e. fiber axis) of the S2 layer have been elusive.

The limited knowledge available on the 3D biopolymer structure of wood and plant secondary cell walls reflects the previously available techniques to investigate ultrastructural characteristics of fiber cell walls, with most of the current knowledge having been derived from indirect biochemical analysis as well as 2D imaging. A multiarray of techniques has been applied for plant cell wall studies ranging from negative staining^22^, surface replica and metal shadowing in combination with transmission electron microscopy (TEM)^23,24^, immuno-labelling/immuno-EM^25,26^, rapid-freeze deep-etching (RFDE)^24,27^, scanning electron microscopy (SEM)/field emission-SEM^14,17,28^, Cryo-SEM^29^, scanning probe-/atomic force microscopy^30,31^, X-ray diffraction^32^ including application of advanced spectroscopy such as Fourier-Transform Infrared (FT-IR)- and Raman spectroscopy^19,33^, wide-angle X-ray (WAXS)-/small-angle neutron scattering (SANS)^12,34^ and solid-state ^13^C NMR^35,36^. These multifaceted approaches have been utilized to address different aspects and/or some of the detail information on wood fiber walls. However, each has their inherent drawbacks sometimes delivering non-collaborating data^34^ reflecting challenges in characterizing the complex biopolymer wood cell wall ultrastructure. For example, resolving the crystalline structure of elementary cellulose fibrils and/or MF aggregates remains difficult and MF diameters (i.e. elementary fibril) are reported with a range of 2.2 to 3.6 nm suggesting differences in the number of cellulose chains between 12 and 32^12,13,37^. This has been debated for decades with the number of cellulose chains within a MF from older studies suggested as 36 while recent work suggest 18–24^13,34,37,38^.

Advances in imaging technologies in recent decades have provided a dramatic change revolutionizing the science of cell biology. For example, with the emergence of electron tomography (ET), that captures 3D information of volume objects from a single specimen, it is now possible to reconstruct cells and organelle subvolumes at “molecular” resolution bridging the gap between atomic resolution defined by X-ray crystallography and global cellular imaging by light microscopy^39–41^. ET has the ability to probe structures at nanometer resolution and the only current method available for providing detailed views on the 3D nanostructure of intact pleomorphic biological objects by computationally combining 2D TEM images of the object. For example, it has been used to visualize and understand sub-cellular components/supramolecular assemblies including their cellular mechanisms in cell biology with ca 2–8 nm resolution in all three orthogonal directions^42–44^. ET has been applied in a variety of biological systems including plant biology^42,45–48^ as well plant/wood fibre cell walls^8,43,49–51^.

However, knowledge on the macromolecular organization and/or biopolymer nano-architecture is limited and unknown in 3-dimensions (3D) despite its great importance in the fields of biology, chemistry, energy and engineering. For example, there remains a gap in our current understanding of the variations in biopolymer nano-distribution, nano-pore architecture and its influence on permeability, nano-mechanical property characteristics and simulations etc. in particular for the dominant S2 layer of mature xylem fibers. The exclusive power of ET can unravel those gaps providing innumerable contributions to the understanding of physical and chemical properties of wood fibers.

In this study, we employed dual-axis ET to investigate wood fiber cell wall nanostructure with emphasis on the dominant S2 secondary layer of Norway spruce fibers. In order to minimize sample preparation artefacts to the macromolecular structure using conventionally processed samples^8^, we employed cryo-immobilization using high pressure freezing (HPF) and freeze-substitution (FS) followed by resin embedding of fresh xylem samples for room temperature (RT) ET. Our approach allows for the first comprehensive study on the 3D macromolecular nano-structure of lignified secondary cell walls of Norway spruce. The study unravels biopolymeric nano-architecture in 3D at a resolution unseen previously (ca 2 nm) and includes simulation of mechanical properties using tomographic data giving novel insights into the structure-related nano-mechanical characteristics of mature wood fiber cell walls. We constructed detailed computational models to visualize and analyze nano-mechanics of the S2 using the data obtained from the density-threshold segmented volume of the 3D reconstructed secondary wall. By fitting prototypic computer-generated models with experimental maps, idealized models containing the measured native architectural design of the original tomogram were created using homogenization-based simulation and model building. The analysis enabled measurements and visualization of nanoscale mechanics across all three directions including stiffness and stress/strain distribution under loading.

## Results and discussion

### Electron tomography, tomogram and segmentation of the spruce S2 wall layer

**Figure 1:**
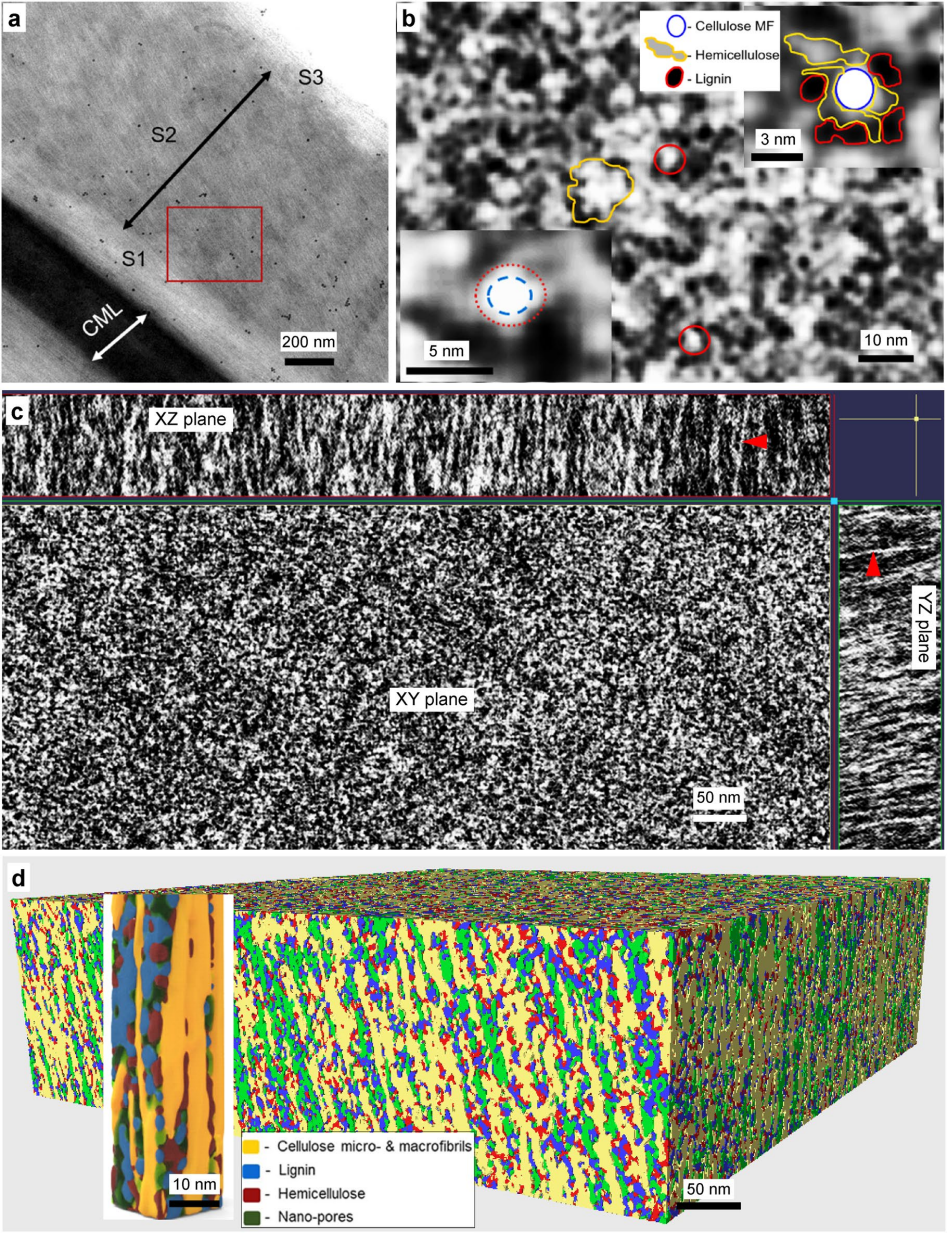
Electron tomography of never-dried mature Norway spruce xylem secondary fiber cell wall for generating 3D macromolecular nanostructure: (**a**) 2D TEM micrograph showing part of a xylem fiber wall illustrating typical cell wall ultrastructure with major cell wall layers (i.e. S1, S2 and S3) after treatment with fiducial markers (black dots). The red rectangle represents the ROI of S2 layer subjected to tilt-series acquisition for producing the tomogram; (**b**) Tomographic slice image of S2 (XY plane) showing cellulose MFs and lignin–hemicellulose matrix network with single (red circles) and aggregates of cellulose MFs (yellow scribble line; white structures grouped together (i.e. macrofibrils)) and surrounded by hemicellulose (grey material) and lignin (black material). Inset top right (legend denotes different grey scale biopolymer structures) shows contour tracing of the arrangement of the biopolymers and inset bottom left showing an example illustrating bright white core (blue dashed circle) of a MF surrounded by a less bright/grey thin layer (red dot circle); (**c**) Part of zap window from IMOD showing x, y and z planes of a S2 tomogram showing arrangement of filamental structures (i.e. straight cellulose MFs; red arrowheads) within a S2 volume and the surrounding matrix polymers; (**d**) First-ever 3D reconstruction and visualization of spruce S2 wall after segmentation showing 3D macromolecular nano-architecture with closely packed biopolymers and nano-pores. Inset shows close-up view of the compact network of S2 biopolymers with hemicelluloses (red) intimately associated with cellulose MFs (yellow) as short structures, lignin (blue) as relatively large structures filling the gaps in the cellulose-hemicellulose network and remaining areas, the nano-pores (green).

The secondary S2 layer of a xylem fiber cell wall from the sapwood of Norway spruce was imaged at 25,000x (Fig. 1a) taking a series of images during data acquisition with a binned pixel size of 0.88 nm ensuring the Nyquist theorem for the highest resolution of the targeted object. Thus, the smallest features of the secondary wall (e.g. individual microfibrils sometimes called ‘elementary cellulose fibrils’)^37^ in our dual-axis tomogram were visualized giving a practical resolution of ~ 2 nm in the X–Y plane.

Mild delignification carried out using peracetic acid before heavy metal staining allows stains (uranyl acetate (UA), potassium permanganate (PP) and lead citrate (LC)) to penetrate inside the fiber wall as a result of a slightly open ultrastructure due to spaces created between the cellulose MFs and MF aggregates^8^. Peracetic acid treatment is an established very mild delignification laboratory method that has high selectivity for lignin removal with very little effect on cellulose and hemicelluloses^52^. The treatment enhanced the visualization of the major cell wall polymers reflected in the contrast between the features in the tomogram. Residual lignin and hemicellulose molecules appeared as darkly (black and grey structures) stained structures while the cellulose MFs were visible as bright white structures as the crystalline cellulose remained unstained (Fig. 1b,c).

Accordingly, the cell wall polymer features and distribution were discernible in the tomographic images and thus could be segmented into the three major wall polymer structures. As shown in Fig. 1b and inset top right, we interpreted the whitest features as the cellulose MFs (red circles in Fig. 1b) and their aggregates (yellow scribble line in Fig. 1b) and the darkly staining materials existing between and/or surrounding white structures (i.e. cellulose fibrils) as lignin (i.e. black structures) and hemicellulose (dark grey) (inset top right in Fig. 1b). This interpretation for the features in the tomogram was supported by the fact that cellulose MFs, which are filament-like structures, were arranged as white (non-staining) structures almost parallel aligned to one another and continuous for a certain distance from the top to bottom along the 140 nm thick tomogram characterized by a nearly uniform diameter (arrowheads in xz and yz planes; Fig. 1c). In contrast, darkly stained lignin and hemicelluloses appeared as non-filamental structures and sometimes as blobs of dense materials which were discontinuous and irregular in shape. These structural features were consistent with those reported previously^8^.

The threshold segmentation tool in Amira 3D visualization software was initially used in semi-automatic mode, which allowed the user to effectively define features of interest manually selecting a threshold for the three polymers. This produced a segmented map, which separately identified three polymeric structures including the S2 layer micro/nano-pores that remained unsegmented after segmenting the three polymers. 3D visualization of the macromolecular nano-architecture of the Norway spruce S2 layer was then achieved utilizing a mesh surface generated from the map of the selected 3D sub-volume of the tomogram.

Dual-axis ET provided the data sets from which reconstruction, visualization and investigations of the 3D organization of the S2 layer of Norway spruce fiber cell wall and its complex 3D macromolecular biopolymeric nano-architecture could be first ever-visualized (Fig. 1d).

### Nano-structure and macromolecular architecture of spruce xylem fiber cell wall S2 layer

High magnification views from different directions of a sub-volume from the tomogram (i.e. xy, xz and yz surfaces) of spruce fiber provided detailed information on the macro-molecular nano-structure of the S2 layer where the arrangements/architecture and distribution of wall polymers were elucidated (Fig. 1b,c). Elementary cellulose fibrils and their aggregates were arranged almost parallel to the vertical axis (xz, yz planes, Fig. 1c and d). MFs appeared to contain an unstained bright white core of ca 2.4 nm diameter surrounded by a mildly stained surface layer (ca 0.7 nm) giving MFs of 3.1 nm diameter (inset bottom left in Fig. 1b). As proposed by Xu et al*.*^8^, postulate the crystalline region of cellulose MFs is thought to represent the unstained white core while the para-crystalline region of MFs corresponds to the lightly stained outer surface layer that we interpret as containing mainly hemicelluloses based on staining characteristics (less bright/light grey outer layer surrounding the middle core; inset bottom left in Fig. 1b). Cellulose MFs were observed as straight filaments with hardly any visible bends or kinks (arrowheads in xz, yz planes in Fig. 1c) although their outer surfaces were not smooth. The uneven surface presumably reflected a heterogeneity in non-crystalline and hemicellulose blend thereby contributing to the difference in diameter at sub-nanometer range along the MF. Available hydroxyl groups of non-crystalline cellulose on the surface of the MFs as well as hemicellulose molecules bound to the non-crystalline region are likely responsible for the heavy metal staining^8^ thereby contributing to the observed mild staining reaction.

In contrast to Xu et al*.*^8^, who showed kinked cellulose MFs in their 3D model of pine secondary cell wall, we did not observe similar deformations along single MF or MF aggregates that appeared as straight filaments (Fig. 1c). Xu et al.^8^*,* postulate that different steps during conventional sample preparation as well as beam induced “thinning” of plastic sections may be responsible for the MF kinking. However, studies using cryo-preserved samples of plant cell walls (e.g. rapid freezing and deep etching (RFDE)^24,53,54^, HPF/FS^50^) show no indication of kinking suggesting such structural distortions of MFs are likely artefacts caused during conventional sample preparation in contrast to cryo-fixed samples. Cryo-immobilization is well known to preserve biological structures at near-native state through vetrification maintaining the original fine details of the sample^55–57^. It is also well documented that resin embedding combined with cryo-fixation and FS can markedly improve the preservation of ultrastructure^56,58^.

HPF/FS is a sample preparation and preservation method for TEM that is particularly useful for studies of thick biological samples (i.e. up to 200 µm thick) including cells and organelles and has been used to address various biological questions^56,59,60^. Work by Sakar et al*.*^50^ showed HPF-FS as an excellent choice for cell wall studies due to consistent high quality and exquisite preservation of cell wall architecture of primary cell walls. Room temperature (RT)-tomography of HPF-FS-resin sections is a relatively easier method shown to generate comparable ultrastructural preservation of primary cell walls compared to technically far more challenging electron cryo-tomography of cryo-fixed cryo-sectioned of vitreous samples. However, the application of HPF-FS in fiber cell wall studies is very limited^50,61^, although Sakar et al*.*^50^ applied HPF-FS on developing young xylem cells from 3–4 weeks old *Arabidopsis* plants. Our work utilizing HPF-FS therefore represents the first reported study with cryo-immobilization and RT-tomography on lignified xylem fibers from a mature trunk of spruce for visualizing the 3D nano-organization of the secondary cell wall.

### Using tomogram for determining the microfibril angle (MFA) of the S2 layer

**Figure 2:**
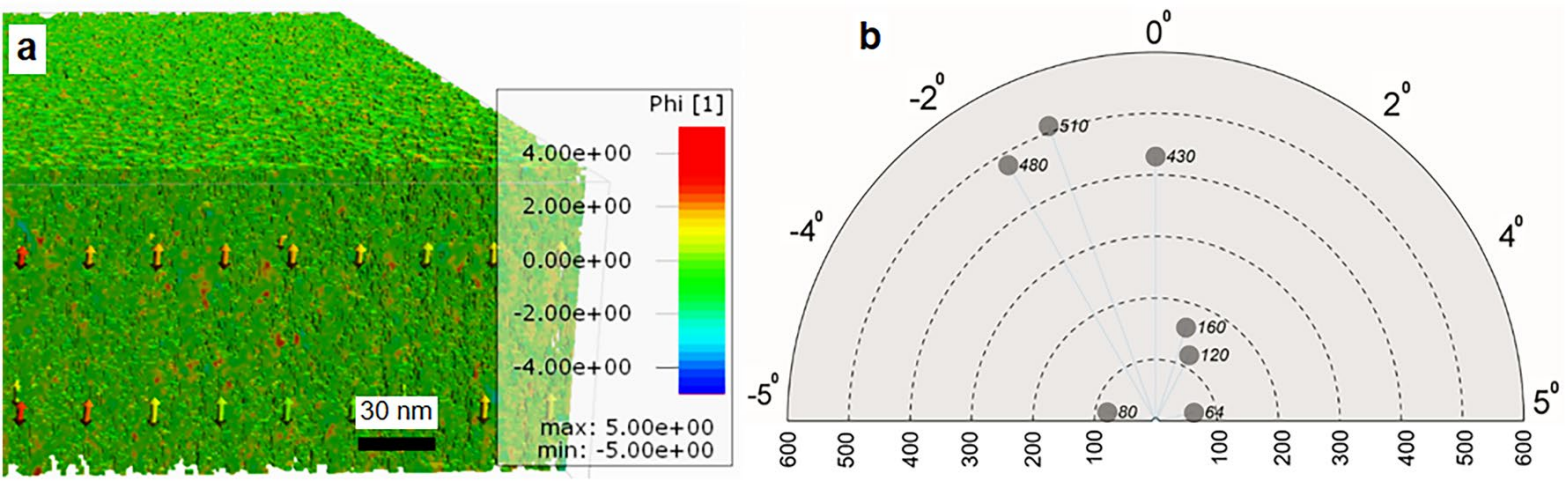
Determining the cellulose microfibril angle (MFA): (**a**) Detailed-map of part of the S2 tomogram showing variations in MFA with colour codes. The map allows each fibril angle in the volume to be traced; (**b**) Summary of MFA measurements within the S2 sub-volume showing microfibril oscillation along the vertical axis, with almost all cellulose MFs at angles between + 5 to − 5°. + /− sign refers to side of the MFA located with reference to Z-axis, i.e. either to the left or right side of the vertical axis. Values on the horizontal axis refer to number of fibres in 100 s for each major dotted line.

Figure 2a,b shows MFA analysis of a given volume of the S2 layer (i.e. 500 × 500 × 140 nm (x, y and z directions) as described in materials and methods) from spruce fiber. Results provided a detailed map (Fig. 2a) that allow tracking of MFs and quantification of the MFA and its distribution (Fig. 2b) within the selected S2 volume. The MFA varied within a short range oscillating between − 5 and + 5° along the vertical axis. The majority of cellulose MFs (< 90%) were at 2° angle (Fig. 2b), which is characteristic of the low MFA found in latewood spruce fiber cell walls. Results agree with previous studies on the S2 layer of spruce latewood fibers utilizing X-ray diffraction^62^ and with Raman scattering^63^. While the former work shows 0° MFA as a mean value over more fibers, Gierlinger et al.^63^ estimates with average values in the range 0–11° from a single latewood fiber.

There is a rich literature concerning MFA and its influence on the properties of wood and fibers where a wide range of techniques have been applied to measure the MFA (e.g. polarization microscopy, soft-rot decay cavities, iodine precipitation or confocal- and fluorescence microscopy, transmission ellipsometry, Raman- and near infrared (NIR) spectroscopy, SEM, TEM, X-ray diffraction). While measurements can provide comparable results and average values and/or overview of a broader area of practical relevance, measured values of MFA by individual methods can vary widely even for a given sample or cell type raising questions on the accuracy^20,21^. Each method is thought to possess associated intrinsic drawbacks including differences in sample preparation, indirect estimation, and influence given by different wall layers, each with a large difference in MFA^21^. In contrast to previous studies, in the present work, the MFs are visualized directly and can be tracked at high magnification.

The present analysis offers improved accuracy with each MF measured visualized permitting tracking of the measurements. Once the tomogram is generated, the current method can automatically analyze and the MFA quantified within a given volume of any of the cell wall layers. In contrast with traditional techniques, the present method (a) considers each cellulose MF organized in a few nms apart within the cell wall volume under investigation, (b) has the potential for quantifying different cell wall layers (S1, S2 etc.) separately evading the influence of adjacent layers, (c) generates a detailed map showing each MF orientation, and (d) provides information on the distribution of MFA with percentages of total measured revealing variability in the MF arrangement in the longitudinal fiber axis. The approach seems therefore promising and could represent a significant change in the way MFAs can be measured offering a complete image of cellulose MF/MF aggregate nano-arrangements across the fiber wall compared with traditional methods such as X-ray diffraction.

### Size (diameter) distribution and nano-architecture of MF and MF aggregates in spruce S2 layer

**Figure 3:**
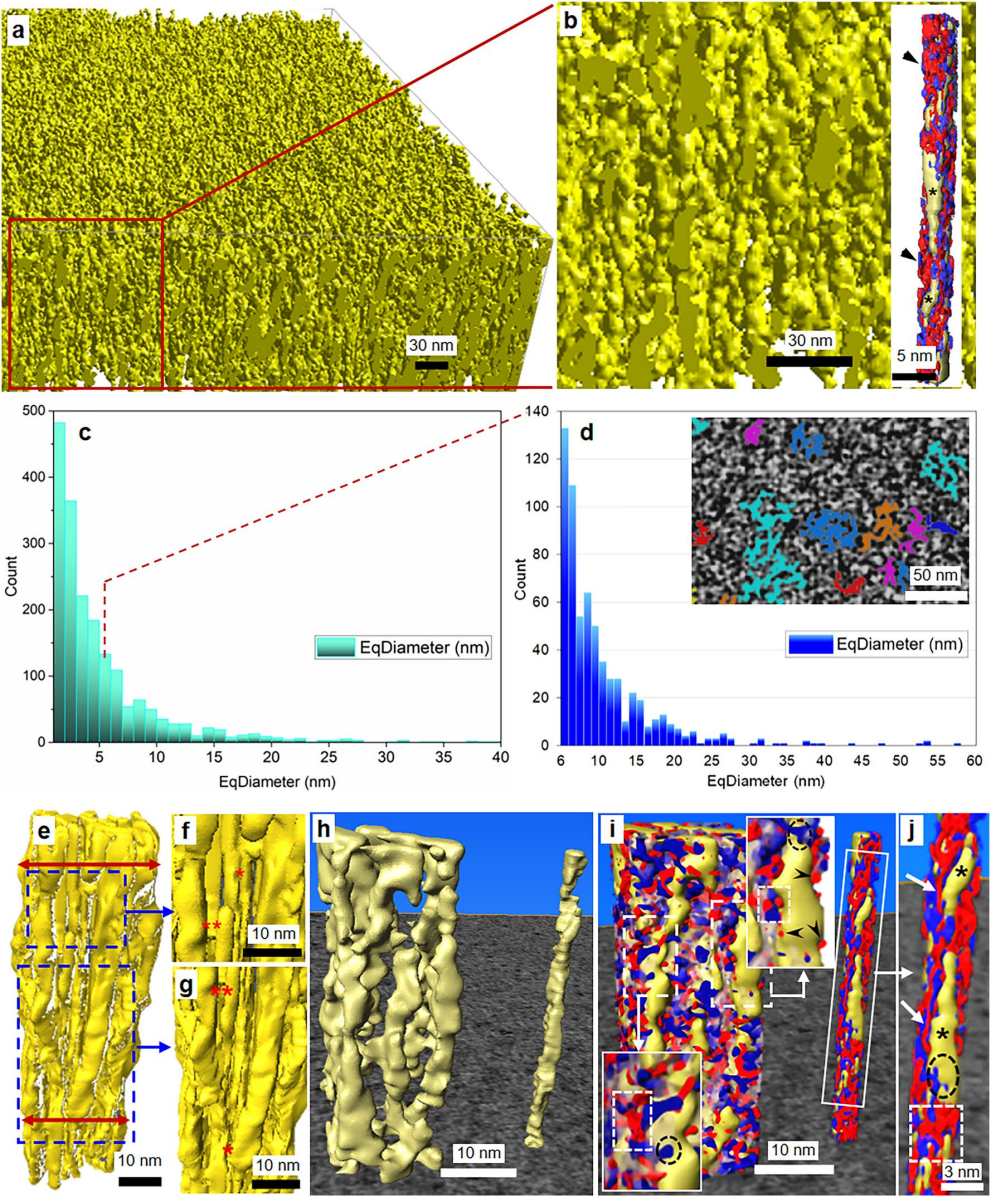
3D nano-organization and nano-features of the cellulose fibrils and matrix polymers in S2: (**a**) Part of S2 showing only the cellulose scaffolds and the 3D arrangement of densely packed fibrils without matrix polymers; (**b**) Close-up view (from (**a**) marked in red box) showing random arrangement of cellulose fibrils. Inset shows a straight microfibril (no kinks) with a blend of hemicellulose (red structures) and lignin (blue structures) inhomogeneously coating the fibril surface along z axis: some regions with only cellulose surface exposed (stars); (**c,d**) Quantifying cellulose fibril diameters and distributions with single MF of 2–4 nm forming > ½ the total fibrils (**c**). Heterogeneity existed among macrofibrils (**d**) with diameters ranging from 6–58 nm with the largest (ca 50 nm) in very few numbers. Inset in (**d**) shows macrofibrils of > 20 nm diameters (coloured areas), largely irregular in cross-section; (**e–j**) Macromolecular nano-architecture of a single MF and macrofibril with only cellulose (**e**–**h**) and with embedded matrix (**i**–**j**). Note change in macrofibril diameter (**e** and** h**) along the length (top and bottom double headed arrows in (**e**)) resulting from differences in MFs packing patterns along z axis. Close-up views (**f**–**g**) from parts of the macrofibril (dashed boxes in** e**) illustrating MFs packing density within the cluster: some regions closely packed (single star in** f** and** g**), while in some places they are separated from the adjacent MF (double stars in** f** and** g**). Note differences in nano-features of surrounding hemicellulose (red structures) and lignin (blue) encrusting macromolecules on the cellulose fibril (yellow filaments) surfaces (Figure** i** and** j** which is part of a single MF from (i) marked in white box). Insets top and bottom in** i** (parts from the macrofibril surface marked in dashed boxes) show structural features of (i) hemicelluloses over the surface of clusters mainly as globules (arrowheads, top inset), (ii) hemicelluloses on single MF primarily as liner ribbon-like structures (arrows in** j**), (iii) lignin–hemicellulose interaction on the surface (dashed box in insets and** j**) and (iv) lignin also directly associated with cellulose fibrils (dashed circle in insets and in** j**).

Results of the cellulose fibril (i.e. MFs and MF aggregates) organization in 3D including matrix polymers, diameter size and their distribution are shown in Fig. 3a–d. The cellulose fibrils which represent the load bearing filamental scaffolding structures of fiber cell walls can be seen densely packed within a given volume of the S2, with few sizable areas devoid of fibrils (Fig. 3a,b). Individual MFs and MF aggregates in the S2 wall existed in fairly similar proportions and were distributed randomly across a given cell volume, with single MFs of 2–4 nm diameter contributing slightly more than half of the total number of cellulose fibrils (Fig. 3c). Furthermore, the MFs showed a rather uniform diameter along their longitudinal axis, apart from the mildly stained outer part that showed variable densities without any obvious pattern attached to the MF surface (Figs. 1b,c, 3b and inset in b). The extremely thin outer region appeared irregular with partially ordered cortex likely composed of a para-crystalline surface of cellulose fibrils (stars; inset (a randomly taken MF from the tomogram with its matrix polymers) in Fig. 3b) and blend of hemicellulose/lignin moieties (arrowheads; inset in Fig. 3b).

The value for MF diameter using ET (i.e. 2–3 nm) was in general agreement with previous studies on softwoods^8^ and recent in-depth analysis on spruce MFs using small-angle scattering (i.e. 2.5 nm)^64^ and spectroscopic methods (i.e. 3.0 nm)^34^. The latter concludes that the surface of MFs with both hydrophilic and hydrophobic faces exposed, contains disordered cellulose chains that are water-accessible and engage abundantly in outward-directed hydrogen-bonding reflecting possible interactions with other secondary cell wall components (e.g. water, hemicellulose and lignin). These interactions with MF surfaces should increase the apparent MF diameter along its longitudinal axis and it has been shown that the mean thickness of the outer surface varies through this disorder as well as by the amount of hemicelluloses bound to hydrophilic faces^34,65,66^. These findings corroborate with present results revealing a non-uniform, irregularly arranged and variable-shaped outer thin surface of the MFs reflecting such a disordered para-crystalline outer surface layer involving a likely blend with other polymers (inset in Fig. 3b).

There was also a heterogeneity in size among the aggregates in the S2 with the majority (ca 96%, Fig. 3d which is an expanded part from Fig. 3c representing whole right area from the dashed line, i.e. starting from 6 nm on the x-axis) ranging between > 6–25 nm diameter in a given cell wall area. MF aggregates form when single cellulose MFs make lateral contacts with each other and where the cluster is encrusted by lignin–hemicellulose matrix^8^. The largest clusters in the present study existed in very few numbers and were slightly greater than 50 nm (Fig. 3d). Unlike single MFs, aggregates were largely irregular in cross-section (Fig. 1b and inset, 3d). This suggests a random aggregation of individual MFs during cell wall biosynthesis creating a non-circular form of large cellulose structures. Measurements from the present study were similar to previously reports on softwood cell walls, with average values ranging between 15 and 30 nm. The MF aggregate diameter is known to vary significantly depending on tree species, cells from different tissues as well as cell wall types with some studies reporting MF aggregates in softwood cell walls up to 60 nm diameter^2,15,67^. Detailed information on size distribution within the secondary S2 layer, similar to present analysis, has however remained scarce. Such heterogeneity in cellulose fibril widths across spruce secondary walls further complicates the already known complex nature of the fiber cell wall, although the reason behind this variation is unknown^2^. Previously established hypotheses for the differences include a relationship where aggregate diameter is proportional to the degree of lignification^15^. Their variability in a given cell wall is likely regulated during cell wall biosynthesis (e.g. density differences of CesA complexes)^68^ and may even be influenced by environmental conditions at the time of biosynthesis^69^.

In the present work, the diameter analysis was done on single 2D planes of the tomogram (i.e. a XY horizontal tomographic slice image) although the value varied along the longitudinal axis of a given aggregate as shown in Fig. 3e (top and bottom double-headed arrows). Individual fibril aggregations were clearly visualized demonstrating how loose and/or close MFs were when packing within clusters (Fig. 3e–h). Their packing character varied along the aggregates (z-axis) revealing differences in the spacing between MFs in a cluster with an average distance of ca 1–2 nm apart. This contributed to the apparent overall diameter variance longitudinally resulting from partial coalescence of MFs where some segments of MFs had direct MF-to-MF contact (single star in Fig. 3f and g) while other segments were separated (double stars in Fig. 3f and g). These findings suggest the potential for water molecules to diffuse along spaces between individual MFs within an aggregate.

Similar irregular spacing was reported in pine fibers with ET^8^ and present results also corroborate with the findings by Fernandes et al*.*^34^ using complimentary FTIR/SANS techniques. The latter study gave unexpected results with evidence of water molecule penetration between MFs within an aggregate to a certain distance upon hydration and the accessibility of water towards outward-facing hydroxyl groups on MF surfaces. Fernandes’ data indicates separation of MFs within a cluster to about 1 nm at 25% D_2_O and finds probable irregular spacing between the MFs. The visualized 3D structure of cellulose fibril aggregates was therefore in line with the findings from this work. However, our findings integrated with these results contrast with previous understanding of solvent inaccessibility of cellulose surfaces within such aggregates because of early ideas for greater “fibril-to-fibril” contact and short contact distances^70^.

Reconstructed 3D macromolecular nano-architecture of single MF and a MF aggregate with- and without surface encrusting hemicellulose and lignin polymers are shown in Figs. 3h–j. The results provide insights into nano-arrangements and blend of the cell wall polymers in the mature fiber S2 layer. The nano features of matrix polymers over the surfaces between MFs and MF aggregates appear to be different (Fig. 3i,j). Hemicellulose macromolecules on the outer surface of aggregates were observed as rather small nano-structures of mostly globular appearance closely associated with cellulose (arrowheads; inset top in Fig. 3i). In contrast, hemicelluloses attached to MFs were seen as large structures of mostly linear (flattened ribbon-like; arrows in Fig. 3j) tightly coating around and along the MF, but with much less lignin (Fig. 3j). The discrepancy may reflect the negative influence on the bonding potential and chemical interaction given by adjacent coalescing MFs in the cluster. We showed longitudinal variations in the spacing between adjacent MFs within a cluster that presumably also contributed to the observed difference in hemicellulose interactions. Interestingly, from the 3D models, lignin was also found directly associated with the surface of the cellulose fibrils (dashed circular marks; top and bottom insets in Fig. 3i and j) in addition to the lignin–hemicellulose interactions existing on the fibril surface (dashed rectangular marks; top and bottom insets in Fig. 3i and j) in contrast to the common understanding on lignin interactions. Lignin has previously been thought primarily as binding to xylan forming a complex in the matrix between MFs but spatially separate from cellulose. Thus, there is a less likelihood for direct contact of lignin with cellulose fibrils in softwood fiber cell walls^71,72^. Furthermore, a significant part of the surface of a MF along its longitudinal axis was coated and blends with matrix polymers although there were some patches where only cellulose surfaces were exposed (stars in Fig. 3j). Due to the lack of heavy metal stains that can specifically bind with different hemicelluloses (i.e. xylan and GGM) in the S2 layer of softwood fibers and thereby give contrast, the present study could not differentiate between mannan and xylan. However, it is likely that both GGM (ca 20% dry wt. in softwoods) with a significant ratio and also xylan (ca 8% dry wt. in softwoods) may contribute to the observed large hemicellulose structures over the MF surfaces based on recent evidence for their interaction with cellulose^65,66^ questioning earlier models^71,72^.

It is only recently recognized that the crystalline arrangement of glucan chains in an elementary MF can generate surfaces with both hydrophilic and hydrophobic faces exposed facilitating selective interaction with different matrix polysaccharides and adjacent MFs^34,65,66,73^. Furthermore, recent work shows that xylan adopts a two-fold screw conformation as well as evenly spaced substitution (i.e. glucuronic acid and arabinose side branches) pattern in coniferous xylan. The new conformation of xylan macromolecular structure in softwoods is also shown to render them with an unsubstituted face that strongly influences their binding to hydrophilic faces of cellulose^65,66,74,75^. In contrast, GGM is the only acetylated hemicellulose in conifers and when highly substituted/acetylated binds poorly to cellulose than less branched GGMs^76^.

Interestingly, a very recent study on spruce identifies the majority of xylan with two-fold screw binding to cellulose fibril surfaces as well as GGMs. The latter also adopts a similar two-fold screw-like conformation when binding to MFs forming flattened ribbons on the MF surface^65^. Using scattering data, Terrett et al*.*^65^ also revealed that a large proportion of both hemicelluloses coats the surface of the same MF and some of the xylan and GGM lie very close to each other within a 5–10 Å distance when bound to cellulose indicating colocation on the MF surface. Using NMR/DNP (dynamic nuclear polarization) techniques, Kang et al*.*^77^ provides evidence showing flat-ribbon structures of two-fold conformers of xylan coating cellulose MFs surface which was also suggested by Granthem et al*.*^75^. In addition, spruce lignin is found tightly associated with both of these cellulose-bound xylan and GGMs as well as some lignin directly to the cellulose surface^65^. These recent findings agree with our 3D visualization of the macromolecular nano-architecture of cellulose fibrils; for example, the linear flattened ribbon-type hemicellulose coating visualized over MFs in the current study (inset in Fig. 3b and j). Terrett et al*.*^65^ did not visualize the 3D nanostructure, instead analysed spectral data for these polymer interactions with solid-state NMR in never-dried spruce cell walls. However, our present study visualized in-muro biopolymer interaction and distribution in 3D over, and along cellulose fibrils with results broadly consistent to their scattering results but are easier to comprehend.

Present study provides insights into the nanoscale information on single MFs as well as MF aggregates. Results provided heretofore-unseen cell wall biopolymer 3D organization of spruce secondary S2 layer with interesting and novel nano-structural details concerning cellulose fibril size distribution, packing characteristics of cellulose aggregates and their nano-architecture blending with matrix polymers. These findings may have a significant impact on future studies (e.g. cell wall biosynthesis, fiber modification etc.) and help to comprehend highly complex macromolecular 3D nano-organization of the major secondary S2 layer including other layers of fiber cell walls.

### Cell wall material distribution across spruce S2 cell wall layer

**Figure 4:**
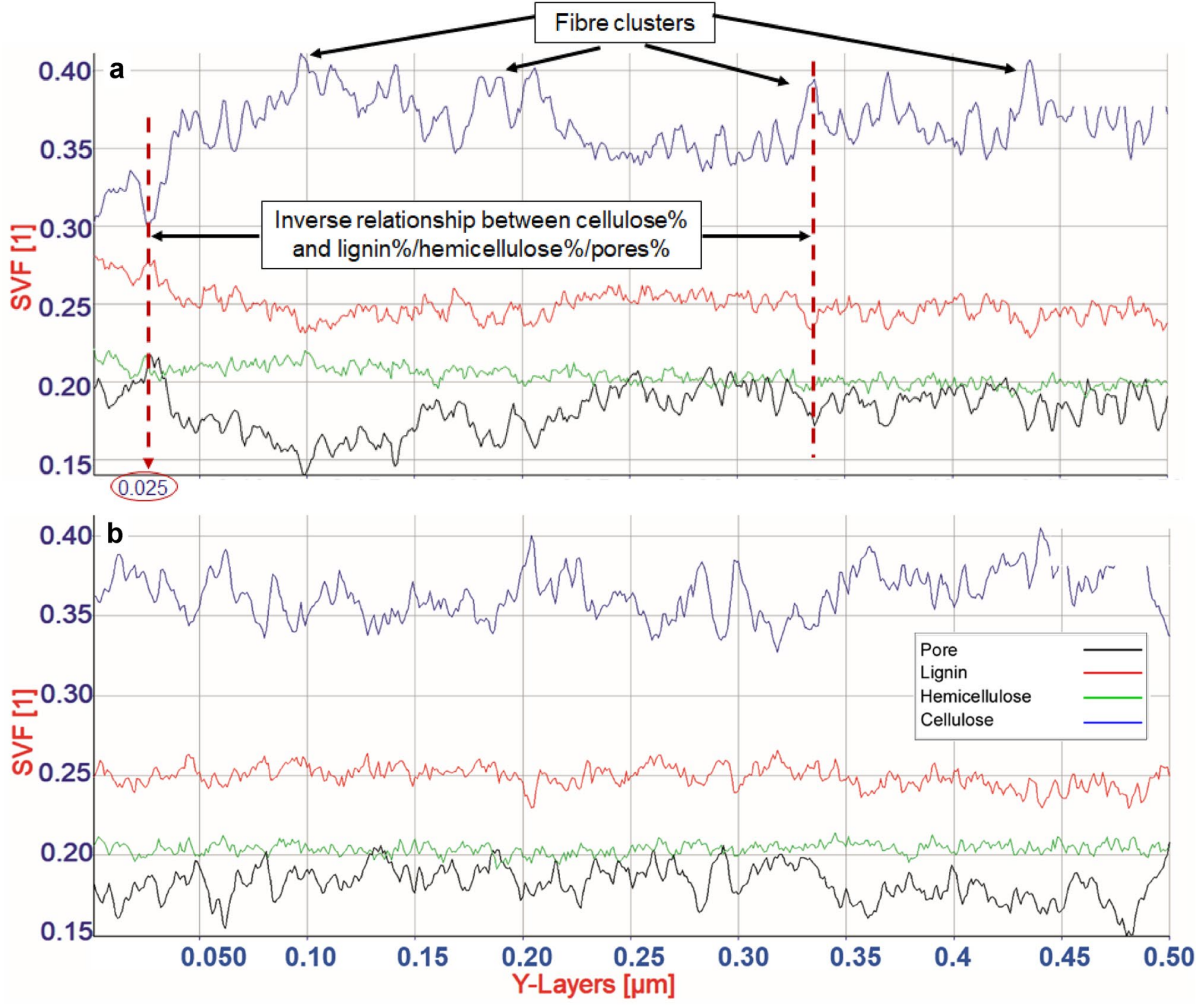
Quantification of chemical composition, polymer distribution and nano-pores in a given solid volume of S2. Chemical composition of the three polymers is spatially highly heterogeneous in both x- (**a**; radial) and y (**b**; tangential) directions of the S2 layer even at nano-meter distances. The percentage of matrix polymers (lignin/hemicellulose) including nano-pores (ca 16% of total volume) follow an inverse relationship to cellulose (vertical broken-lines in** a**).

Quantification of biopolymer composition without chemical extraction, porosity and their spatial distribution using solid volume fractions of the three polymers in the S2 were for the first time investigated. Figure 4a,b illustrates heterogeneity in the spatial distribution of cell wall biopolymers at the nano level within a 500 nm range of the tomogram analyzed across X (Fig. 4a) and Y (Fig. 4b) directions. The overall chemical composition (as solid volume fraction) of the major biopolymers of the S2 was found to contain ca 33–43% cellulose, ca 21% hemicellulose and 23- 26% lignin with the rest (11–20%) non-solid unfilled fraction representing the pore structure of the S2 layer. Clearly visible was the dynamic variation in cellulose in both radial/tangential directions since cellulose exists in axial filaments and in clusters that are separated with the drop in cellulose content occurring between fibrils. Relatively broad high peaks in the graphs represent cellulose clusters. Further percentage variation of matrix polymers was relatively low, even though the porosity changed rapidly compared to that of the matrix polymers. Most striking was the percentage of matrix polymers (i.e. both lignin and hemicellulose) and their spatial distribution in any given region that follows an inverse relationship to cellulose with the cell wall porosity following a similar pattern (dashed vertical lines in Fig. 4a). The spatial variation in lignin was also prominent with a clear opposite change to cellulose content at a given point when compared with hemicellulose. The percentage drop in cellulose did not often compensate with the percent rise in lignin (e.g. the change at the region 0.025 µm on the x-axis; left dashed arrow pointing the value in Fig. 4a) presumably contributing to the formation of pores. This supports the fact that lignin is primarily responsible for filling the gap between cellulose fibrils and thereby pore formation as the hemicellulose fluctuation over the entire region analyzed was mild and remained more or less constant as shown in Fig. 4a,b.

The porosity of S2 also appeared to vary upon cellulose change. However, the variable increase in apparent porosity (upon reduction in cellulose), despite an increase of predominantly lignin deposition at the same space, suggests an in-homogeneous and incomplete lignification when filling spaces after cellulose fibril biogenesis and hemicellulose deposition. This would eventually lead to the formation of pore structures in the native fiber S2 layer through incomplete filling of inter-microfibrillar regions (Fig. 4a,b), generating a random 3D network of pores. For example, at the cell wall region 0.025 µm along the x-axis in Fig. 4a (left dashed arrow), there was a drop in % cellulose by about 3 units (i.e. 34 to 31%) but the drop was compensated by only < 2 units (26 to ca 27.8%) of partial filling of the space by lignin leading to an empty space (i.e. represented by only resin in the section) as the change in hemicellulose was minimal. This has not been visualized previously, although there has been similar hypotheses based on indirect analysis using gas adsorption^78–80^. Donaldson et al*.*^81^ reported indirect observations using confocal microscopy on cell wall porosity of wood that are also consistent with present results demonstrating porosity fluctuations. Using fluorescence microscopy probes, they assessed the porosity of native cell walls of different cell types and in different cell wall regions concluding more lignified cell wall regions are generally less porous with lignification likely determining cell wall porosity of xylem cells. Our findings thus reflect the nature of biopolymer biogenesis. Once cellulose fibrils are synthesized in a cell wall region, there is limited space available to deposit matrix biopolymers, thus the reduced proportion of both lignin and hemicellulose, with the former likely determining porosity of lignified cell walls.

The chemical composition obtained in the current study on a given solid volume from unextracted native fibers without laboratory chemical analysis was comparable to results on spruce wood from traditional wet chemical analyses. Values obtained in-muro were in the range previously reported by pure chemical analyses generally yielding (w/w) ca 41% cellulose, ca 25% hemicellulose and ca 27% lignin^16^. Here, in-muro refers to the native location of wood biopolymers including S2 pores, in contrast to extracted in vitro cell wall chemistry. Changes in cell wall polymer concentrations from one morphological region to another is well known, but present results quantifying a non-uniform distribution of polymers even within small cell wall regions with concentration changes radially and tangentially at nano-meter distance across the S2 is new. Even though the unfilled nano/micro-pores included here as a fraction in the total influence the estimated absolute chemical values, slightly lower amounts of matrix polymers observed with lignin were likely the result of mild delignification during sample preparation. Compared with in vitro chemical analysis that also offers other chemistries such as extractives, ashes etc. the present approach provided pore distribution as a fraction which is otherwise impossible to determine without utilizing separate physicochemical methods such as porosimetry, gas adsorption, or freezing point depression^80,82^. 

The current tomographic approach on solid chemistry provides an excellent foundation for future research on lignocellulose fibers for insights into in-muro biopolymer biosynthesis, biomineralization, distribution/re-distribution during fiber processing, fiber modification and biofuel/bioenergy applications. It not only quantifies bulk chemical composition in a given volume but also measures spatial variations in chemistries in all three directions even at nano-meter distances, unlocking a potential for deriving profound chemical information even for a minute variations in each layer across the fiber wall in-muro. No such methodology is currently available that provides detailed analysis of chemical component variations in 3D of lignocellulose fibers. A drawback however is current limitations with extractive and ash analyses including different hemicellulose moieties (e.g. GGM and xylan), but advances in imaging technologies (e.g. electron energy loss spectroscopy (EELS) and energy-filtered TEM (EFTEM)) will likely help to overcome such limitation in the near future^83,84^.

### 3D nano-pore structure of the S2 layer of native spruce fiber wall and its permeability

**Figure 5:**
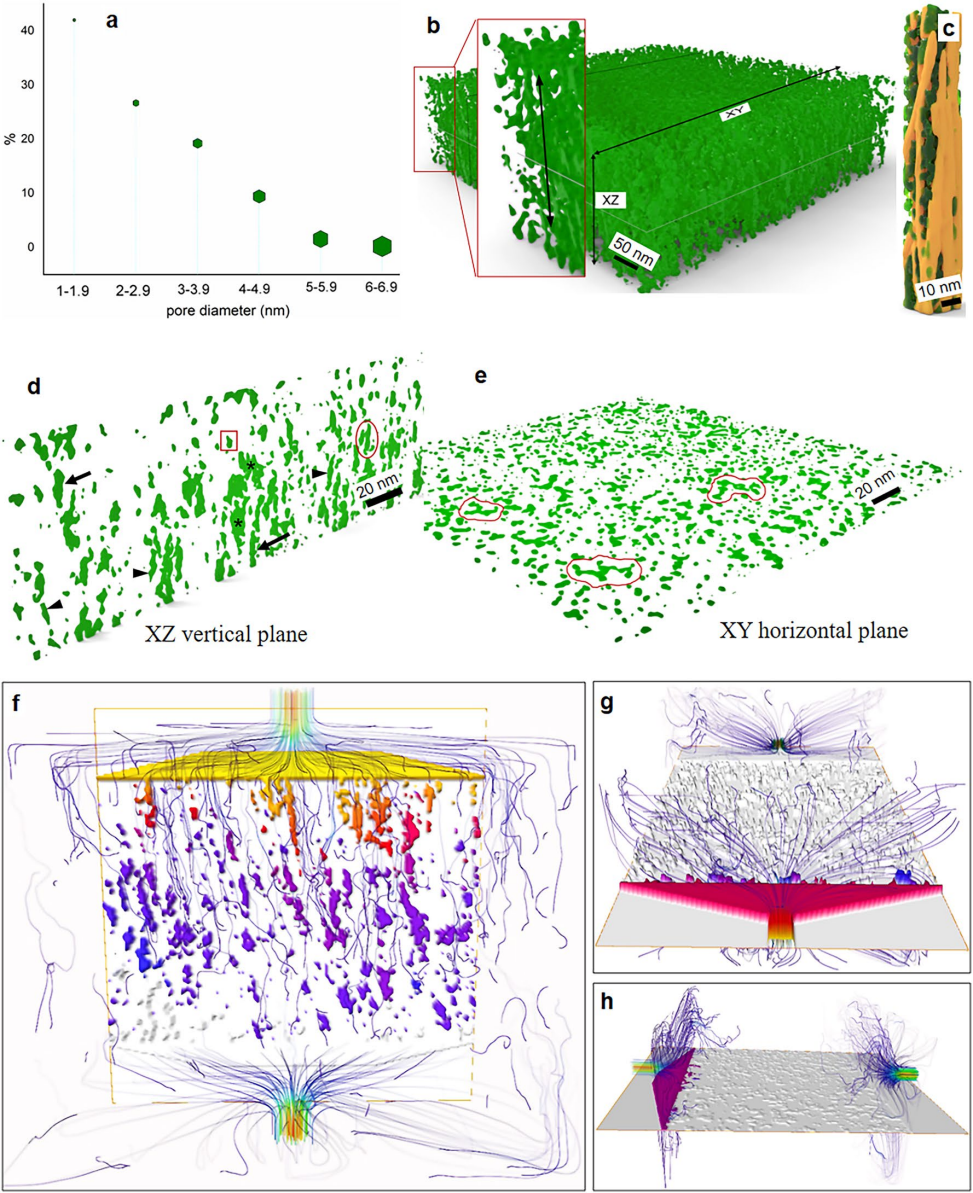
Nano-porosity (ca 16% v/v) distribution, nano-pore architecture and permeability characteristics of the S2 layer: (**a**) Summary of measured pore diameter distribution where the majority of pores (ca 70%) was < 3 nm; (**b–e**) Native nano-pore architecture (without biopolymers) in 3D (**b**), within a macrofibril (only cellulose MFs with pores, without matrix polymers, are shown) showing axial arrangement of pores among single MFs (**c**), pores visible from longitudinal side (**d**, xz- vertical plane) and transverse (**e**, xy-horizontal plane) views. Pores of the native fiber S2 layer follow the cellulose fibrils running along the z-axis (axial arrangement indicated by double headed arrow, inset in** b** and** c**-**d**) in an interconnected network giving different geometrical shapes like tubular/cylindrical (arrows in** d**), slit-shaped (arrowheads, in** d**), wide pond-like (stars in** d**), horizontally interconnected tubular pores (circle, in** d**), single/closed pores of ‘inkbottle’-shaped (red box in** d**) etc. In contrast, pores visible on the transverse plane (**e**; xy horizontal plane) differed in geometry (e.g. circular/oval to inkbottle/slit-shaped and irregular pores) and networking features with no apparent pattern (scribble line markings); (**f–h**) Results of permeability simulations showing water movement after pressure set on transverse (**f**; water flow both horizontally and along the fiber axis), radial (**g**) and tangential (**h**) directions of the tomogram/cell wall. Fluid flow trajectories within S2 are visualized with streamlines that represent the flow regions according to the local velocity (i.e. velocity fields) which was superimposed on pressure field. The default blue-to-red colour isosurfaces were used to map the pressure fields with blue representing low pressure and red for high pressure. Note the significant differences in water flow in the three directions of the fiber wall with greater flow along the longitudinal axis.

Pores are generally classified as micropores (< 2 nm diameter), mesopores (2–50 nm) and macropores (> 50 nm) with the porosity of wood fiber cell walls thought to exhibit at molecular dimension both micro- and mesopores^82,85^. However, since the present study provided values of pores in diameters of the order of nanometers, technically they should be referred to as ‘nano-pores’. Here, we report the 3D architecture of nano-porosity and details of nano-pore structure of the S2 layer of spruce fibers at an unprecedented level (Fig. 5). As shown in Fig. 5a, the pore structure of the S2 layer was primarily composed of nano-pores within the matrix polymers in inter-microfibrillar regions (inset in Fig. 1d). The total nano-porosity (v/v) within a given volume of the S2 was 16% (Fig. 4a,b) which was assumed to be slightly higher than in the native S2 wall due to mild delignification (discussed below), and pore size distribution is shown in Fig. 5a. Equivalent diameter of almost all nano-pores (> 98%) was less than 5 nm and ca 70% below 3 nm diameter with the majority of the latter pores < 2 nm.

Present results are consistent with previous findings on pore diameters of xylem fiber walls where entire cell walls or whole tissue were investigated using indirect approaches. Previous methodologies are very different to our approach that allowed direct visualization of 3D pore structure and measurements targeting only the S2 layer. It is known that different techniques for measuring fiber wall pore size provide different values and are dependent upon the state of the wood fibers (e.g. dry or wet). Nevertheless, it has been established from numerous studies that the maximum pore size of never-dried wood fibers lies in the region of 2–4 nm with some reports of diameters no greater than 2 nm corresponding well with present results^79,80,82^. Yin et al*.*^85^ reports comparable results on the pore structure of fiber walls in Chinese fir. The greater proportion of pores have 1–4 nm diameters with a peak of 1.4 nm pores in heartwood fibers in comparison to the highest percentage of pores at < 2 nm size observed from the present study in spruce (Fig. 5a).

Here we performed very mild delignification of samples to improve heavy metal penetration into the fiber wall for enhancing TEM contrast among the biopolymers. This was expected to loosen the dense cell wall structure and slightly remove some lignin and possibly a little hemicellulose allowing the stains (UA, LC and PP) to infiltrate deep inside the wall into spaces in the interfibrillar regions and stain lignin and hemicelluloses^8,86^. Therefore, the cell wall will be slightly ‘open’ creating new pores in the wall. The native pore structure was thus expected to slightly change providing some increase in the total pores than in the native wall. Pore formation in fiber walls is heavily influenced by its chemical composition and lignin is reported to be inextricably linked with cell wall porosity. Lignin is found associated with nanopores smaller than 0.6 nm and delignification can modify the porous structure causing enhanced mesopores in the range 2–10 nm^82,85,87^. The inter-relation between lignin and porosity of lignified cell walls is also supported from results by Donaldson et al*.*^81^ who provides evidence for a relation existing in heartwood vs. sapwood and softwood vs. hardwood.

Our observations emphasize the important role played by the nano-pore structure of the dense and tightly packed S2 wall layer of lignocellulose fibers, in particular during enzymatic treatments in many applications (e.g. biorefining, biofuel, wood/fibre impregnation, pulping and bleaching etc.). The most commonly known lignocellulose degrading enzymes are much larger (e.g. cellulase with ca. 5.9 nm in size) than native fiber cell wall pores measured in the current study hindering penetration and accessibility of the enzymes for the targeted substrate^88^.

Accordingly, the cell wall nano-pore architecture (Fig. 5b–e) was likely the result of incomplete infiltration of matrix biopolymers, mainly lignin, between the natural load bearing elements cellulose fibrils. This is a randomly created network structure within the tightly packed biopolymers and the previously unseen 3D nano-architecture of S2 pores displaying an interconnected network (Fig. 5b–e). When viewing along the longitudinal fiber axis, as shown in Fig. 5b (i.e. marked ‘xz’ longitudinal side), the pores sometimes appear to follow the cellulose fibrils running along the z-axis leading to a somewhat axial arrangement of pores aligning with the cellulose fibrils as depicted in Fig. 5b (double headed arrow in inset) and 5c. In this plane (Fig. 5d), tubular-shaped (cylindrical type; arrows) or extended slit-shaped pores (arrowheads) are commonly visible that may result from interconnected nanopores forming elongated pore structures. Adjacent tubular- or slit-shaped pores can also be seen horizontally interconnected (oval mark in Fig. 5d) while longitudinally arranged ‘pond-like’ large pores (stars in Fig. 5d) existed presumably due to unified adjacent pore clusters. There were some areas without pores inferring an intense packing density of the material while isolated solitary pores were also common. The totally isolated pores were enclosed regions (‘closed-pores’) that were relatively small, mostly oval-shaped, but sometimes also with circular or ‘inkbottle’ shapes (square mark in Fig. 5d) and were dispersed throughout the area under investigation.

In contrast, pores visible on the transverse plane (i.e. in cross-sections of the fiber wall) differed considerably in geometry and networking features from pores in longitudinal view, and no organized pattern of orientation was apparent (Fig. 5e; xy horizontal slice image). Pores in the transverse surface were randomly dispersed and had variable geometries including circular, oval, inkbottle, slit-shaped, interconnected tubular to irregular pores. Both isolated and interconnected pore structures were frequently observed where the latter were rather irregular and branched or unbranched ‘pond-like’ structures reflecting the extended nature of interconnection among adjacent pores (scribble markings in Fig. 5e). These branched pond-like pore aggregates were not as wide as those observed on the longitudinal surface but were narrow linked tubular-like structures in contrast to elongated and broad pond-like pore aggregates viewed in the vertical plane. Unlike the longitudinal view, pores on transverse surfaces were scattered across the entire wall so there were very few sizable areas deprived of pores.

The 3D architecture of S2 nanopores unraveled from the current study is an important phenomenon of the densely packed lignified secondary wall with respect to fluid/water movement and its mechanics in wood/plant fiber cell walls. For such a nano-porous structure, longitudinal water transport or diffusion is expected to be much easier than horizontal or across the cell wall due to the 3D interconnected “anastomosing-type” network apparently existing in the longitudinal than transverse plane. However, the continuation of nano-pores both horizontally or vertically was randomly disturbed after some nanometer distance because of infiltrated matrix polymers. The average basic geometry of single pores seem to be oval and/or slit-like/tubular in shape although irregular and other types are also not uncommon in the S2. Daniel et al*.*^24^ previously showed oval-shaped pore structures in kraft pulp fibers whereas slit-shaped pores are commonly recognized in native fiber walls from Chinese fir evaluated based on hysteresis loop^85^.

Visualization of the 3D nano-architecture of S2 pore structure motivated us to investigate permeability characteristics of the lignified S2 layer with the use of a simulation platform in order to detect the circulatory pattern of water movement throughout the S2 porous space. Assuming a single-phase flow through nano-pores to exist within the solid fraction of the wall (i.e. biopolymers), which is impermeable, the data obtained from the tomogram was used to compute absolute permeability of a given volume of S2 by applying Darcy’s law. As an example, water pressure was set to 130 kPa at the inlet (cross-section; Fig. 5f), radial (Fig. 5g) and tangential (Fig. 5h) direction of the cell wall/tomogram. Results suggested that water becomes frequently trapped and the flow is terminated after travelling some distance within the pores along the vertical axis of the fibers (Fig. 5f). Here, water movement occurred both horizontally and then vertically reflecting native nano-pore network structure revealed in this study. Water could not freely travel throughout the porous space along the fiber axis although there were certain regions where water can travel without encountering obstacles (Fig. 5f). Nonetheless, permeability along the z-axis (i.e. fiber axis) of the wall is much greater compared to transverse water movement, i.e. via the radial (Fig. 5g) or tangential (Fig. 5h) direction of the fiber wall with the latter more superior to the former (Fig. 5g vs. h).

These simulated experimental observations on water transport through the lignified wall corresponded with the visualized 3D nanopore structure explaining, at least partly, permeability results. Axially arranged and interconnected nanopores provided pathways and thereby facilitated water transport/diffusion along the fiber axis and the axial flow was terminated at the end of the space that was clogged by matrix polymers. Furthermore, the axial arrangement of S2 pores was mainly attributable to the difference in the permeability characteristics between vertical and horizontal (i.e. radial and tangential lateral movements) water flow through the fiber wall. Both these lateral movements were characterized by short lateral diffusion combined with axial movements presumably following continuous axial arrangement of pores. Interestingly, Donaldson et al*.*^81^ reports similar results with in situ experiments on chemical diffusion through xylem fiber secondary walls. It was found that diffusion of dye along a longitudinal surface is much greater than across a transverse surface and suggested, based on indirect assessment, to likely exist directionality with cell wall porosity that possibly align with the cellulose MFs. They however did not visualize but rather suggested pore directionality using indirect investigations while the current study confirmed axial alignment of pores using direct visual evidence.

The present results on the 3D architecture of nano-porosity and associated permeability characteristics along and across the S2 may therefore have a significant impact on future studies on wood and fiber research in particular wood impregnation, chemical pre-treatments, biorefining and pulping.

### Idealized model generation, simulations and bio-nano-mechanical analysis of spruce fiber S2 wall

**Figure 6:**
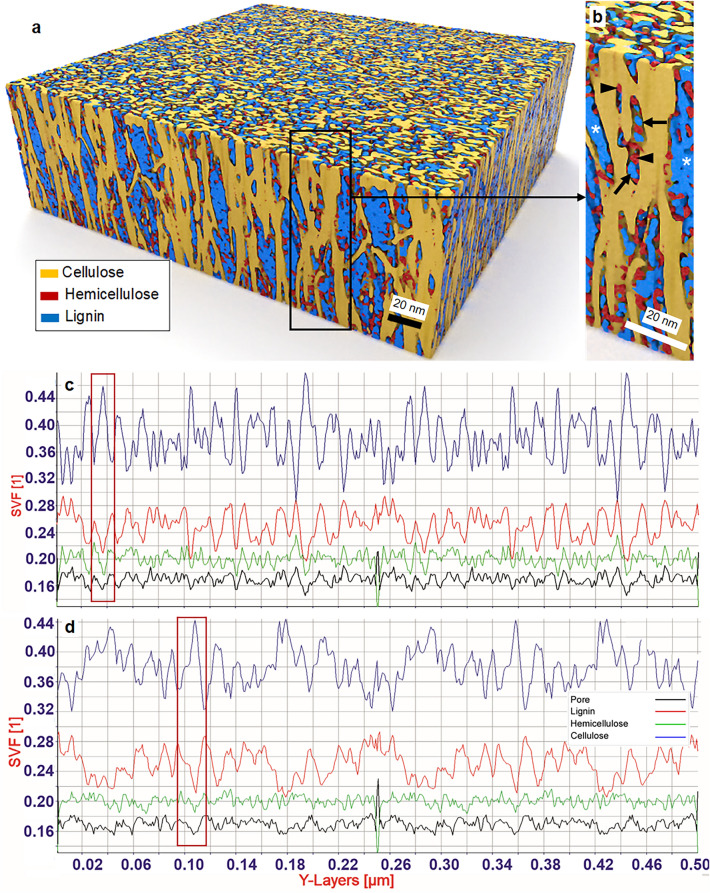
Generated idealized model of the S2 layer based on quantified data from spruce fiber wall tomogram: (**a**) 3D visualization of the idealized S2 model showing similar visual appearance as the 3D reconstructed native S2 layer regarding nano-architecture (cf. Fig. 1d); (**b**) Close-up view showing biopolymer assembly, arrangements and distribution; (**c,d**) Analysis of biopolymer composition (ca 40% cellulose, 20% hemicellulose, 25% lignin on average (v/v)) and spatial distribution along radial (**c**)- and tangential directions (**d**) of the fiber wall. Dynamic variation in spatial distribution showed an inverse relationship between cellulose and matrix polymers including nano-pores (rectangular markings in** c** and** d**) similar to that of the native fiber S2 layer.

The nano-meter quantitative data extracted from the tomogram of the spruce S2 layer inspired the generation of an idealized model of part of the S2 layer using a simulation approach. The overall dimensions of the model generated (Fig. 6a,b) in x (radial), y (tangential) and z (height) was 500 × 500 × 140 nm respectively and the volume contained cellulose MFs with randomly varying of diameter between 2 and 5 nm. Presence of aggregates was simulated by overlapping between cellulose MFs while fibril orientation was allowed to vary randomly between 0 and 5° with the generated idealized model shown in Fig. 6a,b. Of the two major matrix polymers, hemicellulose deposition attached to cellulose fibrils was simulated to infiltrate in a particulate form with globular pattern (arrowheads in Fig. 6b), as observed in the tomogram, having dimensions ranging between 2 and 5 nm and 20% as its solid volume fraction. Lignin infiltration was deposited in the spaces among the cellulose and hemicellulose polymers in the volume (Fig. 6b). Simulated lignin deposition was represented in the tomogram where the particulate form of lignin (arrows in Fig. 6b) was 3–5 nm diameter and where its solid volume fraction reached 25%. However, during the simulation process of lignin assembly, lignin globules frequently merged and deposited into confined interfibrillar spaces (stars in Fig. 6b).

The visual appearance of the biopolymer arrangements and distribution in the idealized model was very similar to the nano-architecture produced in the tomogram of the native S2 layer. Analysis of the idealized model demonstrated a similar material distribution in all three directions x, y and z. Like in the native wall, when the fraction of cellulose is increased at any given point in space, both the hemicellulose and lignin fractions also decreased including the nano-pores (long rectangular marks in Fig. 6c,d). Despite a higher variation in the percentage distribution of biopolymers across X (Fig. 6c) and Y (Fig. 6d) directions compared to that of the native wall, they showed similar average values of material percentage across the three directions; 20% hemicellulose and 25% lignin that filled the void spaces between the cellulose fibrils and associated hemicellulose. This advancement in lignocellulose research with simulation techniques has a great potential for understanding cell wall characteristics and unravel mysteries in wood and fiber behavior during mechanical and chemical modifications, for example mechanical properties (as shown below).

**Figure 7:**
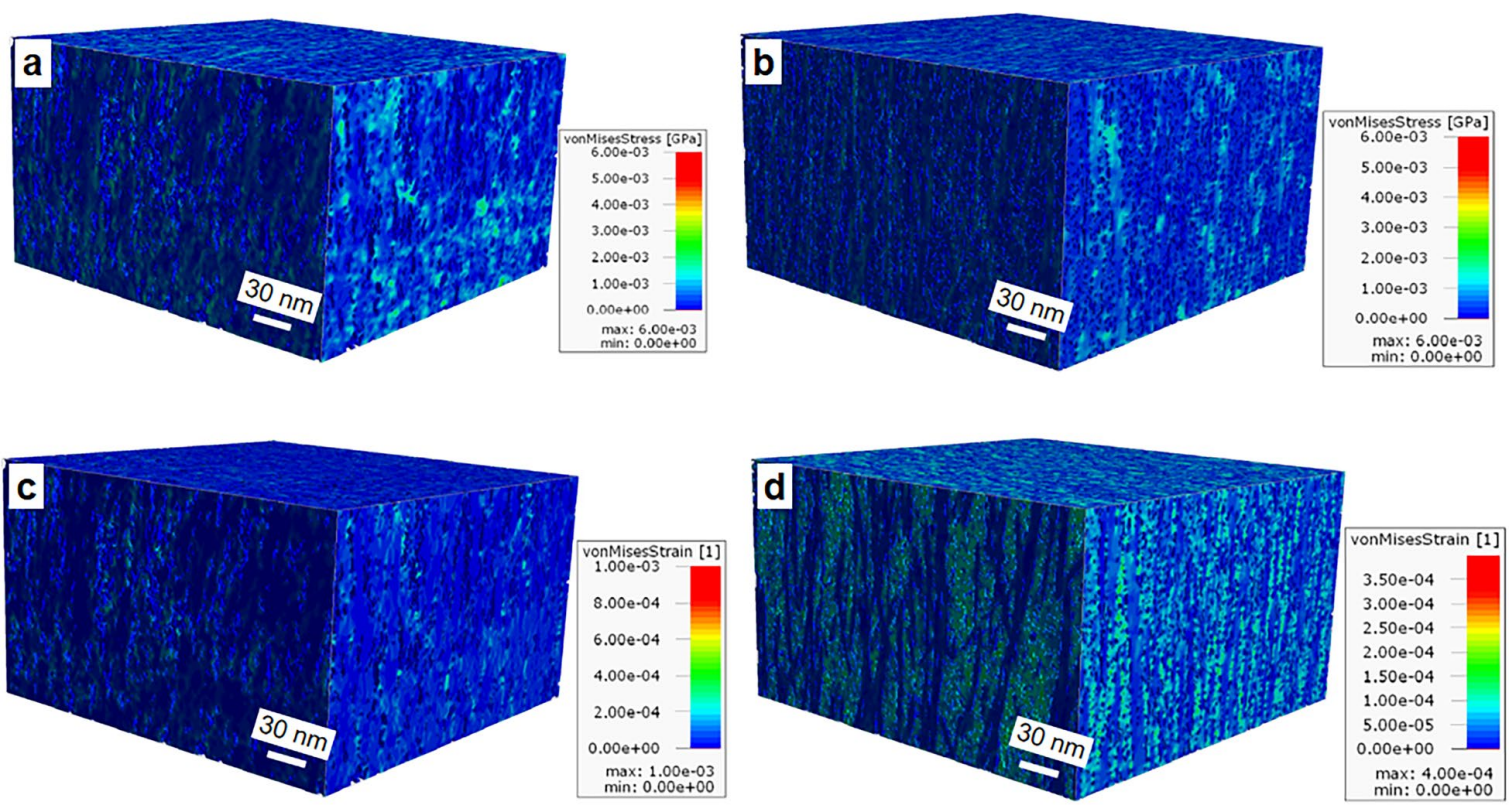
Bio-nano-mechanics of the native S2 wall (a,c) and idealized model (b,d). Spatial distribution of von Mises stress at nano scale in the 3D volume of the S2 layer (**a**) and idealized model (**b**) under six load cases (3 tensile and 3 shear) and the resulting strain on the native wall (**c**) and its simulated model (**d**) where the respective effective stiffness of the material was computed (see Fig. 8). Note the similarities at the nano level distribution of von Mises stress and strain between the two models.

Nano-bio-mechanics of the native (i.e. using the tomogram) and idealized model of the S2 were investigated under six load cases (three tensile and three shear) where the respective effective stiffness of the material was computed in each load case (Fig. 7). The spatial distribution of von Mises stress in the actual 3D volume (Fig. 7a) and idealized model (Fig. 7b) of the S2 showed similar nano-scale distribution within the cell wall. For example, comparable results were observed with respect to relatively high stress concentrations and their distribution in space of a given volume (ca 1.5 E^−0.3^ GPa, Fig. 7a vs. b). However, the resulting overall von Mises strain distribution (Fig. 7c,d) was found to be slightly different compared with the idealized model (Fig. 7d) that showed slightly higher values and better distribution in longitudinal direction parallel to each other implying that strain occurs primarily in the cellulose fibrils. This discrepancy may be attributed to the collective counterbalancing mechanism of biopolymers in native cell walls that may deviate in the idealized model. The contribution of biological association and inter-molecular bonding of the three biopolymers and the resulting shared response to the stress applied may not strictly represent in the simulated idealized model where cellulose fibrils are primarily strained which was probably not the case in the native wall.

Mechanical properties like elastic modulus of plant cell walls vary depending on wood species, age, region of the wood (e.g. sapwood vs. heartwood) and chemical composition, properties of the individual wall constituents as well as the arrangement and properties of the wood polymers^89,90^. Wood is also an orthotropic material exhibiting distinct material properties in three orthogonal directions. For example, the secondary wall of *Arabidopsis thaliana* was found to possess an elastic modulus of ca 59, 25 and 6 MPa for longitudinal (fiber axis), tangential and radial direction respectively^91^. Mature cell walls of spruce are much stiffer and known to have transverse orthotropic characteristics meaning radial and tangential elastic moduli are similar compared to the longitudinal modulus which is much higher^62,89^.

**Figure 8:**
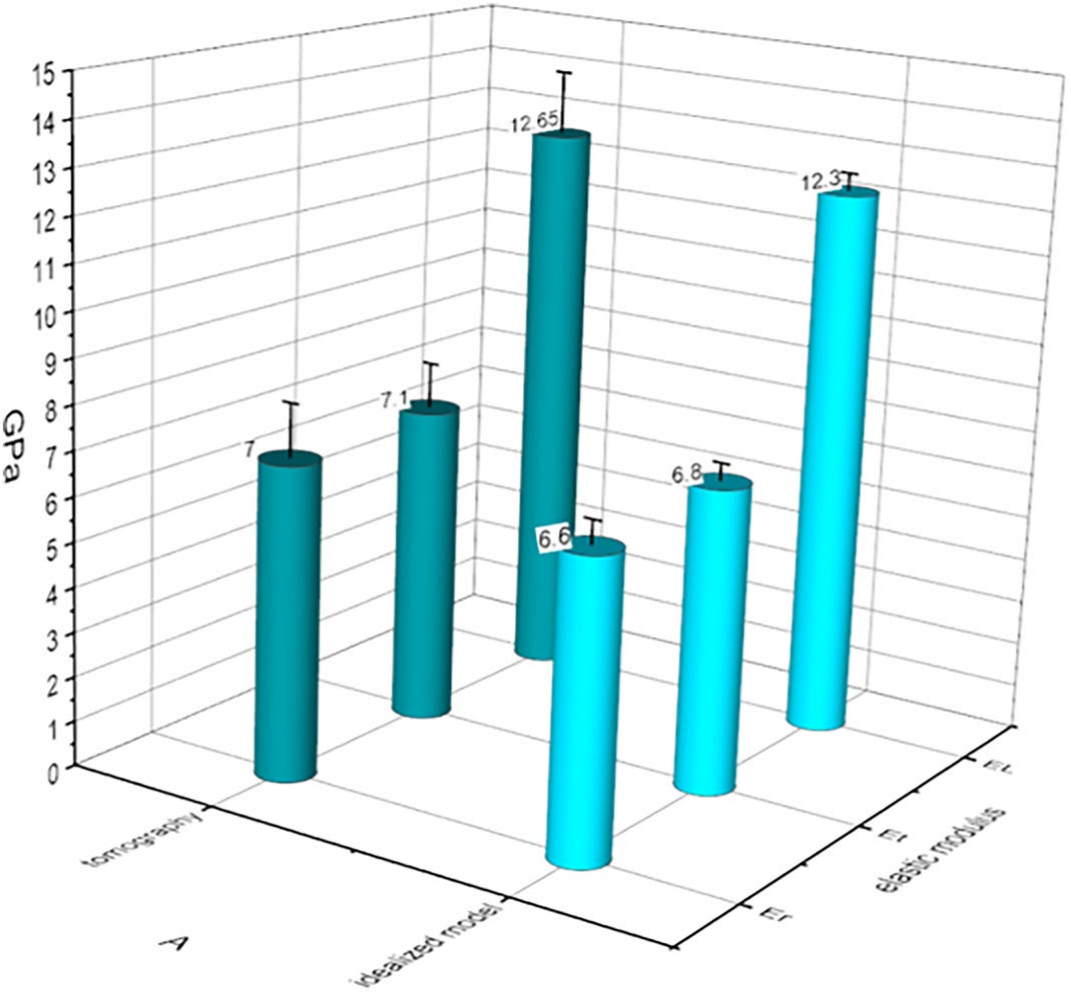
Summary of the computed elastic moduli of the native wall and its idealized model. Both exhibited transverse orthotropic behavior where the longitudinal modulus was almost twice as stiff as the radial/tangential elastic modulus and showed similar nano-mechanical properties in all three directions of the fiber wall.

In the current study, the elastic moduli of the spruce S2 layer for both native (i.e. part of the tomogram analyzed from the S2) and idealized models in all three directions were estimated at nano-scale (Fig. 8). Results indicate that the orthotropic character of macro-scale wood materials exists at the nano-structure level of the S2 layer which exhibited typical transverse orthotropic behavior; with the longitudinal modulus (E_l_; 12.65 GPa) ca twice as stiff as the radial (E_r_) and/or tangential (E_t_) (ca 7 GPa each). Interestingly, the nano-mechanics of the 3D reconstructed native S2 layer and its idealized model show almost similar nano-mechanical properties. The longitudinal modulus for native wall was 12.65 vs. 12.3 GPa for idealized model, while radial and tangential moduli were 7 vs. 6.6 GPa and 7.1 vs. 6.8 GPa respectively. This is a significant finding providing greater opportunities with a major change in the direction of wood and fiber research with respect of wood/fibers as engineering materials.

## Conclusions

Present methodology employing ET of cryo-immobilized lignocellulose fibers allowed visualization of the 3D macromolecular organization and biopolymer (i.e. cellulose, hemicellulose and lignin) nano-architecture of spruce fiber secondary S2 wall layer revealing previously unseen nano-details including quantitative analyses. Single cellulose microfibrils (MF) of 2–4 nm diameter were arranged (i.e. MFA) between + 5 and − 5° in the vertical axis while MF aggregates (macrofibrils) showed heterogeneity both in morphology and size distribution along and across the aggregates of up to 60 nm diameter. 3D nano-arrangements and blend of matrix polymers encrusting single MF and MF aggregates were unraveled showing differences over the surfaces between the cellulose fibrils with lignin interpreted as directly associated with cellulose in places in addition to lignin–hemicellulose interactions. In situ biopolymer quantification provides a novel way for future research on lignocellulose chemistry providing information on cell wall pores and their spatial and temporal 3D variation in a non-destructive manner. The amount of matrix polymers including porosity at any given point, radially or tangentially, followed an inverse relationship to cellulose while lignin showed oscillation upon cellulose change indicating its influence on the formation and design of pore structure in the S2 wall. Heretofore unseen 3D nano-architecture of S2 nano-pores, the majority of which were < 3 nm diameter, were visualized and quantified with major differences in geometry and networking features found between horizontal and vertical planes where the latter showed pores aligned longitudinally with the cellulose fibrils. Simulated permeability characteristics of the densely packed S2 wall followed its nano-structure that influenced liquid flow in the S2. Nano-biomechanics of the native S2 and idealized 3D model showed similar values on elastic moduli between the two 3D models. They provided nano-scale stress–strain concentrations and distribution showing the presence of orthotropic character in nano-structure of the dominating S2. Our approach utilizing electron tomography on lignocellulose biomass opens up new possibilities for plant fiber research for 3D nano-scale characterization and quantification bridging various aspects of the biomass into a common platform while offering in-depth understanding on 3D structure–property relationships of the biomaterial down to nano-level.

## Materials and methods

### Sample preparation

Fresh Norway spruce (*Picea abies* (L.) H. karst) xylem tissues obtained from a mature tree located near SLU, Uppsala (N 59.81°, E 17.66°, (WGS84)) were used for the study. The plant material was collected from the SLU University campus close by the Department laboratory at which part of the experiments was carried out. Both the collection of the material and the research complies with institutional, national and international guidelines. The plant material was collected from a non-restricted area open to the general public and appropriate permission has been obtained for collection of plant material.

Small pieces (2 × 2 × 1 mm) of never-dried xylem samples were first subjected to very mild delignification using peracetic acid for 4 h at 70 °C^8^, washed in water and ca 200 µm thick hand sectioned xylem samples high pressure frozen in a HPC-010 HPF (BAL-TEC, Balzers, Liechtenstein) with 1-hexadecene as cryoprotectant. The frozen samples were freeze-substituted in a mixture containing 0.5% glutaraldehyde + 0.1% tannic acid in anhydrous acetone at − 90 °C for 24 h, followed by three acetone rinses (15 min. each) at − 90 °C and 24 h incubation in 1% w/v OsO_4_ + 0.1% w/v uranyl acetate (UA) in acetone at − 90 °C in a Balzers FSU 010 (BAL-TEC, Balzers) freeze substitution apparatus. Specimens were then slowly brought to room temperature over a 24 h period and rinsed three times with acetone (15 min. each). The samples were removed from the holders (HPF planchette) for resin infiltration at room temperature as follows: incubation in 50:50 Epon:acetone overnight, replaced with 100% uncatalyzed Epon for 1 h, followed by 100% Epon with catalyst for 1 h (× 2) and polymerization in 60 °C for 2 days. Resin-embedded samples were sectioned at ca 140 nm thickness using a diamond knife and sections collected on copper slot grids coated with formvar support films. Care was taken to cut ultra-thin sections at 90° to the fiber longitudinal axis. Sections were post-stained for wood holocellulose as follows: 12 min. 2% w/v UA, 6 min. lead citrate (LC)^92^, 4 min. potassium permanganate (PP) (1% w/v in 1% w/v sodium citrate), and 4 min. LC^8,86^. Sections were thoroughly washed between all staining steps. All sections were exposed to gold fiducials (10 nm diameter), for 4 min. each on both sides of the grids, followed by washing in distilled water. The fiducial markers were used for alignment of the tomogram. After careful examination under TEM for a suitable area for tomography, the S2 secondary cell wall of the xylem fibers (from a latewood tissue area) was used for data acquisition.

### Data collection

A Tecnai F20 FEG TEM (FEI, Eindhoven, The Netherlands) was used operated at 200 kV with digital images recorded via a Gatan 2Kx 2 K CCD camera during tomographic data acquisition. Dual-axis tilt series were collected by tilting the specimen in the microscope from − 60° to + 60° with 1° increments. Once the first tilt series were recorded around axis A, the specimen was manually rotated 90° for the second tilt series around axis B. Images of the raw stacks were collected using a binned pixel size of 0.888 nm.

### Data processing: image alignment, 3D reconstruction/visualization and generating the 3D macromolecular nano-structure

IMOD software (The Boulder Laboratory for 3D Electron Microscopy of Cells, University of Colorado Boulder, CO, http://bio3d.colorado.edu/imod/) was used for alignment and initial tomogram generation. We reconstructed the 3D volume from each axis individually (i.e. volumes A and B) before combining them into a final tomogram of the 3D volume from part of S2 wall layer.

Segmentation, 3D structure visualization and generation of the 3D macromolecular nano-architecture of part of the S2 wall layer were performed using the 3D visualization software Amira Ver.6.4 (Thermo Fisher Scientific, Berlin, Germany). Before initiating the segmentation process, noise reduction and contrast enhancement of the tomograms was performed using image filtering techniques (e.g. deblur, bilateral filter and contrast enhancement). The semi-automated threshold segmentation approach produces reliable thresholding for unstained cryo- and stained resin embedded samples of plant cell walls^50^, and was applied during segmentation to extract the cell wall features from small 3D volumes of the reconstructed tomogram. During the process, the ‘threshold segmentation’ tool in Amira was used in combination with selecting a threshold value by visual inspection of cell wall features one at a time (i.e. three major grey-scale differences in black, white and grey colours) that was denser and/or different to the surrounding background colour in order to segment the feature of interest. Areas that were not segmented were considered as background and were interpreted as cell wall pores. These areas were interpreted as composed of resin only. The ‘unconstrained smoothing’ filter in Amira was applied to generate triangular mesh surfaces from the segmented images providing improved 3D visualization of cell wall features for generating the 3D macromolecular nano-structure of the selected part of the S2 layer. The unconstrained smoothing filter provides a smoother representation when each surface is created.

### Segmentation, quantitative image analysis and measuring wall features in a given 3D volume of the secondary S2 wall layer

Following 3D visualization, quantitative analysis was performed using GeoDict 2017 (Math2Market, GmbH, Kaiserslautern, Germany) as follows. After noise reduction and contrast enhancement described above, segmentation was initialized by employing unsupervised iterative K-means clustering algorithm to classify voxels into K groups (K = 3) corresponding to three material phases (i.e. cellulose, hemicellulose and lignin) of the wood components. The algorithm applied performed automatic classification of voxels into the three clusters and the whole segmentation process on the tomogram was carried out in GeoDict. Segmented tomograms were used in subsequent data analyses performed on a given volume (x, y and z) of 500 × 500 × 140 nm randomly selected close to top left of the tomogram of S2 layer.

Fibril diameter analysis (i.e. individual microfibrils and microfibril aggregates) was performed in GeoDict. The software computes equivalent diameters (i.e. frequently used in image analysis suites) with results provided in the form of a diameter histogram where the diameter vs fibril volume fraction of that diameter is plotted (as %). Pore diameters were also computed with the equivalent diameter method in GeoDict by analyzing pore spaces in which pore size distribution was used. Pore radius was determined by fitting spheres into the pore volume but the method, which is purely geometrical, cannot distinguish between totally enclosed- or ‘open’ pores. Following the diameter analysis, a permeability experiment was carried out on the cell wall tomogram in order to detect the circulatory pattern of water within the pore space of the S2 layer. The water pressure at the inlet was set to 130 kPa and the solid fraction of cell wall was made impermeable. The absolute permeability was computed with a single-phase flow by application of Darcy’s law^93^ and the experiment performed using Avizo 2019.1 (Thermo Fisher Scientific, Berlin, Germany) software.

MFA (i.e. microfibril orientation) was measured by computing a tensor for characterizing the orientation distribution of the cellulose MFs using the FiberGuess module of Geodict 2017. Briefly, the orientation of the sample was approximated by partitioning it into sub-cubes from where automatic analysis of orientation was done. For each voxel, cord lengths were analyzed for a predefined set of directions with the per-voxel orientation tensor obtained with the relative cord length. The tensors, that describe the statistical distribution of orientation in a particular block, were then averaged over all voxels included in a grid cell. A detailed map revealing fibril orientation was generated and colour coded the cellulose MF orientations (i.e. the MFA).

Quantification and spatial distribution of wall polymers was carried out in Geodict, which calculated material characteristics of the 3D models obtained from the tomographic data. Solid volume fractions (SVF) for each of the three polymers and total non-SVF (i.e. porosity) were measured in three spatial directions. The values are the average for the entire material(s) inside the bounding box. After determining the SVF in the different spatial directions and measuring the material thickness, the SVF is computed in the plane normal to the direction of interest. The average value for each plane is then determined.

An idealized macromolecular model was generated from the S2 tomographic data. This model was influenced by quantified information from the tomographic data including fibril diameter, fibril orientation, polymer concentration etc. The process was initialized with the specified overall dimensions in x, y and z for the model similar to the dimensions of the volume analyzed of the actual tomogram (i.e. 500 × 500 × 140 nm). The idealized model incorporating secondary wall polymers was generated in GeoDict that populated the specified volume with cellulose microfibrils of randomly varying diameters between 2 and 5 nm during fibril deposition process. Overlapping between MFs was allowed to vary randomly thereby simulating the fibril clustering (i.e. macrofibrils) effect occurring *in-vivo* while the fibril orientation (MFA) varied randomly between 0 and 5°.

Computation of the biomechanics of the secondary wall and idealized model through simulation were performed in GeoDict by reading their voxel data. For the S2 wall, the tomogram was split into four regions 250 × 250 × 140 nm to facilitate computations. The effective elasticity tensors of heterogeneous materials were calculated after computing equivalent material properties that required solving six load cases (three compressive and three shear). For each of the selected load cases, the effective stiffness of the material was computed. The presented methodology is applied for estimation of effective properties of the cell wall.

## Data Availability

The datasets generated during and/or analyzed during the current study are available from the corresponding author on reasonable request.
